# Effects of Integrated Chinese Traditional Medicine and Conventional Western Medicine on the Quality of Life of Breast Cancer Patients: A Systematic Review and Meta-Analysis

**DOI:** 10.1155/2022/3123878

**Published:** 2022-01-07

**Authors:** Xue Bai, Na Ta, Guo-Hua Gong, Bin Zhang, Cheng-Xi Wei

**Affiliations:** ^1^Inner Mongolia Key Laboratory of Mongolian Medicine Pharmacology for Cardio-Cerebral Vascular System, Inner Mongolia Minzu University, No. 996 Xilamulun Street (West), Tongliao 028000, Inner Mongolia, China; ^2^Public Health Teaching and Research Office, Inner Mongolia Minzu University, No. 996 Xilamulun Street (West), Tongliao 028000, Inner Mongolia, China; ^3^Affiliated Hospital of Inner Mongolia Minzu University, Institute of Mongolia and Western Medicinal Treatment, No. 1742, Huolinhe Street (East), Tongliao 028007, Inner Mongolia, China

## Abstract

**Background:**

Traditional Chinese medicine has been widely used, in conjunction with conventional Western medicine, in clinical practice around the world to treat breast cancer. The study systematically reviewed and summarized the quality of life of breast cancer patients treated with integrated treatment method vs. conventional Western medicine.

**Methods:**

Eight databases including PubMed, EMBASE, Web of Science, the Cochrane Library, Chinese National Knowledge Infrastructure, China Biology Medicine Disc, Chinese Scientific Journal Database, and Wanfang Data knowledge service platform were searched in this study. The retrieval period was set from January 1, 2005, to December 31, 2020.

**Results:**

Twenty-two high-quality publications were included in this study. The total sample size was 1689 patients including 844 in the intervention group receiving traditional Chinese medicine combined with conventional Western medicine and 845 patients in the control group receiving conventional Western medicine only. Compared with the single-used conventional Western medicine treatment, an integrated approach to treat breast cancer can increase quality of life measured by rating scales (SMD = 1.29, 95% CI (1.07, 1.52) and *P*=0.01) and ranking scales (RR = 1.53, 95% CI (1.39 1.68) and *P*=0.02) and also decrease adverse reactions measured by rating scales (*Z* = 10.89, *P* < 0.05; Group 1: *I*^2^ = 9.0%, *P*=0.258, SMD = 1.03; and Group 2: *I*^2^ = 31.6%, *P*=0.199, SMD = 1.56). For further analysis, chemotherapy with epirubicin exhibited higher quality of life than the chemotherapy without epirubicin among breast cancer patients [*Z* = 19.80, *P* < 0.05; Group 1: *I*^2^ = 62.4%, *P*=0.070, SMD = 1.61; and Group 2: *I*^2^ = 9.0%, *P*=0.359, SMD = 1.04]. Despite the heterogeneity, which was due to a portion of relative low-quality literature or other factors, the results were satisfactory. In terms of secondary results, the patients with lower tumor markers (CEA and CA153) had better efficiency in quality of life with a statistically significant difference (SMD = 1.39, 95% CI: 1.10,1.67) for rating scales. In addition, secondary results related to high incidence of gastrointestinal adverse reactions (RR = 1.33, 95% CI (1.20, 1.48)) and the traditional Chinese medicine syndrome (RR = 1.50, 95% CI (1.28, 1.80))showed lower quality of life in the intervention group than the control group for ranking scales.

**Conclusion:**

Traditional Chinese medicine, when used in conjunction with the conventional Western medicine, could be an effective way in improving the quality of life and alleviating incidence of associated adverse symptoms such as gastrointestinal adverse reactions, value of tumor markers, and the incidence of traditional Chinese medicine syndrome. Further investigation of larger and methodologically sound trials with longer follow-up periods and appropriate comparison groups is needed.

## 1. Introduction

Breast cancer is the most prevalent disease that threatens the health of women globally. Every year, more than two million women in the world are newly diagnosed with breast cancer, accounting for about 25.0% of the total number of all cancers among women [1, 2]. According to GLOBOCAN, breast cancer, among all malignant tumors, has the highest incidence and mortality rate in women [3]. Over the past three decades, China had a high growth rate of breast cancer with 3–5% especially in large cities such as Beijing, Shanghai, and Tianjin, and in the next 20 years, it will be the most severe malignant tumor [4, 5]. Even more, it has been predicted that, by 2021, the cases of breast cancer will reach 2.2 million in China [5].

There are varying perspectives on the etiology, pathogenic mechanisms, and treatment approaches of breast cancer, but there is a lack of unanimous statement due to the different levels of impact. Conventional Western medicine (CWM) proposes that hormonal factors, postmenopausal obesity, nonbreastfeeding, endocrine disorders, and abnormal increases of estradiol and estrone are the mechanisms affecting breast genes and inducing cell damages, which could deteriorate into abnormal breast tissue proliferation and eventually develop into breast cancer [6]. In traditional Chinese medicine (TCM), emotions are considered the main cause of the disease. Emotion causes liver Qi depression, blood and Qi disorders, and Qi-regulating body fluid dysfunction, resulting in the formation of sputum due to stagnant body fluid. A tumor develops if Qi obstruction and phlegm stagnation in breast vessels persist for an extended period. Currently, treatment methods for breast cancer patients are dominated by the CWM treatment, which includes surgery, radiotherapy, chemotherapy, targeted therapy, and hormonal therapy, among others, which are considerably effective in managing the disease and prolonging life [6, 7]. Among them, the most common treatment is chemotherapy using drugs such as Adriamycin, cyclophosphamide, docetaxel, epirubicin, fluorouracil, letrozole, megestrol, paclitaxel, and tamoxifen, which expand patients' life [8]. For example, cyclophosphamide is a nonphase specific cytotoxic agent and commonly used as an anticancer drug [9]. It is composed of a nitrogen mustard group (bis-chloroethylamine) attached to an oxazaphosphorine ring [10]. Another commonly used chemotherapeutic drug is 5-FU that interrupts nucleic acid synthesis. 5-FU is a broad-spectrum antitumor drug used in the cancer clinic including breast, head, gastrointestinal, etc. In 1954, pyrimidine uracil usage was found to be elevated within rat tumors [11]; subsequently, bone affinities were established by the in vitro hydroxyapatite (HA) affinity assay and their cytostatic effects were demonstrated by the MTT test by measuring the inhibition rates on osteosarcoma cells [12]. However, although these methods have the advantage of inhibiting the uncontrolled cell division process for the treatment of different types of cancer [9], there are also serious side effects of these drugs on the hematopoietic system, bone marrow, and gastrointestinal epithelial cells and hair follicles, which are crucial disadvantages [9]. Therefore, a growing body of evidence indicates that although CWM can improve the overall survival of patients, it cannot completely eradicate the disease and is often accompanied by adverse side effects, including a decrease in white blood cells and immunity, vomiting, gastrointestinal symptoms, massive liver and kidney damage, and emotional reactions such as anxiety and despair. This may be due to lack of selectivity for tumor cells, insufficient drug concentration in tumor tissues, drug resistance, and systemic toxicity [13]. Cancer patients suffer from both the disease and effects of CWM, which may reduce the quality of life (QOL), and it has been reported to be lower than other population groups [14, 15].

Early detection of breast cancer and advancements in medical technology and treatment methods have increased survival rates and the number of cured patients [7, 16]. Expectedly, survivors may experience negative psychological and physical symptoms due to long-term reactions and performance in cancer treatment and anxieties about the possibility of recurrence [17]. In addition, previous studies indicated that some comprehensive interventions typically encompass a wide range of psychosocial, behavioral, and environmental strategies that may complement conventional treatment to enhance QOL by alleviating disease side effects and restoring physical functioning with similar survival outcomes for cancer patients [17–19]. The World Health Organization defines quality of life as “an individual's perception of their position in life in the context of the culture and value systems in which they live and in relation to their goals, expectations, standards, and concerns” [20]. Many scales with good reliability and validity have been applied around the world, including the European Organization for Research and Treatment of Cancer Quality of Life Questionnaire-Core 30 (EORTCQLQ-C30), Karnofsky Performance Status (KPS), and the MOS 36-Item Short-Form Health Survey (SF-36), to assess the sexual enjoyment, future perspective, systemic therapy side effects, breast symptoms, and hair loss, among others [21].

Interventions of TCM as an adjuvant method, such as acupoint stimulation, moxibustion, Chinese massage (referred to as “Tuina”), Tai Chi, Qigong, and Traditional Chinese Medicine Five-Element Music Therapy (TCM-FEMT), have been widely practiced and accepted as effective methods for reducing the side effects, minimizing toxicity, reinforcing the treatment effects, and reverting multidrug resistance in clinics [22, 23]. Of the aforementioned traditional Chinese medicine methods, acupuncture involves piercing a needle (usually a filiform needle) into the patient's body at a certain angle under the guidance of the theory of Chinese medicine, and acupuncture techniques such as twisting and lifting are used to stimulate specific parts of the human body to achieve the purpose of preventing and treating diseases like cancer and strengthening body immune resistance [24]; moxibustion involves the application of heat, which stimulates skin thermally by burning of moxa, at precise locations or other specific areas, which enhances blood circulation and relieves swelling and pain; and in Chinese massage, practitioners use their finger, hand, elbow, knee, or foot with a wide range of technical manipulations to muscle or soft tissue at specific body locations [25]. These methods have been accepted and extensively practiced to prevent and treat diseases worldwide since 3500 years ago during the Shang Dynasty of China [26–28]. Based on the above benefits, some studies hypothesize that TCM can improve the QOL by minimizing the adverse effects through lowering cachexia, fatigue, and bone loss while also improving mental health, cardiovascular functioning, muscular strength, and bone flexibility among tumor patients [29].

Based on previous research, the integrated treatment method can enhance the treatment efficacy while alleviating toxicity. However, no evidence-based studies have explored the use of TCM in conjunction with CWM to improve QOL and reduce adverse reactions in breast cancer patients. Furthermore, neither the intervention approach nor outcome assessment has been clearly specified. Differences in study design, intervention types, frequency and duration, and strategy may produce varying results. Therefore, based on the results of previous reliable and effective scales that provide guidance as supportive and adjuvant therapy for the clinical application, we performed a meta-analysis to systematically review the quality of life and related clinical endpoints between TCM in combination with CWM and CWM alone explicitly for treating breast cancer.

## 2. Material and Analysis

### 2.1. Protocol and Study Registration

This study was carried out following the Preferred Reporting Items for Systematic Reviews and Meta-Analysis (PRISMA) guidelines. We registered the study on PROSPERO under the registration number CRD42021231966.

### 2.2. Search Strategy

A thorough literature search was performed in electronic network databases, including foreign databases such as PubMed, EMBASE, Web of Science, and the Cochrane Library, and Chinese databases including China National Knowledge Infrastructure (CNKI), China Biology Medicine Disc (SinoMed), the information resource integration service platform (VIP), and Wanfang Data knowledge service platform (Wanfang Data). The retrieval terms were mainly based on a mix of MeSH subject words and random words.

The type of disease was searched by “Rufangzhongliu OR Rufangai OR Ruyan OR Ruai OR Ruxianzhongliu” in Chinese and “Breast Neoplasms OR Breast Tumors OR Breast Cancer OR Breast Mammary Cancer” in English. The types of studies were limited by searching the Chinese words “Suijiduizhao OR Suijifenzu OR Suijiquzu” or English words “randomized controlled trial OR placebo”. In Chinese, the terms “Zhongxiyijiehe OR Zhongyixue OR Zhongyiyao” were used, whereas in English, the phrases “Traditional Chinese Medicine OR Chung I Hsueh OR Traditional Tongue Diagnoses” were used. The measurement of outcome was searched by “Shengmingzhiliang OR Shenghuozhiliang” in Chinese and “Quality of life OR Health-Related Quality of Life” in English. The retrieval type or blinding was not restricted, and the research was conducted from January 1, 2005, to December 31, 2020.

### 2.3. Inclusion and Exclusion Criteria

#### 2.3.1. Inclusion Criteria

The inclusion criteria of this study were as follows: (1) studies on postsurgery breast cancer female patients who were diagnosed by pathology, cytology, and imaging examination and had no recurrence, metastasis, or evident complications that could influence the outcomes of the current study. There were no age or cancer stage limits; (2) the intervention group was treated with a mix of tradition Chinese medicine and conventional Western medicine, while the control group was treated with only conventional Western medicine; and (3) the primary outcome was health-related quality of life as measured by reliable and validated instruments, with cancer-related performance as a secondary outcome (details of the indicators were shown in “Outcome Indicators”).

#### 2.3.2. Exclusion Criteria

The exclusion criteria of this study were as follows: (1) nonrandomized controlled trial (non-RCT) studies; (2) systematic reviews, case reports, meeting abstracts, animal tests, and commentaries; (3) inconsistent, incomplete, or ambiguous baseline data on the age, disease, and other associated characteristics of the participants; and (4) lack of original data, only a part of useful data presented, or the authors failed to reply upon being contacted.

#### 2.3.3. Intervention and Control Measurements

The intervention group was treated with TCM methods, such as acupuncture, moxibustion, Qigong, and Chinese herbs, combined with CWM techniques, such as chemotherapy, radiotherapy, and endocrine therapy. Among all the TCM methods, the designated acupuncture points were manually manipulated to obtain De Qi, which is numbness, distension, or electrical tingling sensation at the needle insertion sites that may radiate along the corresponding meridian.

The control group received only CWM treatment methods, including chemotherapy, radiotherapy, and endocrine therapy. In our study, we only included CWM as the research approach in control group. The chemotherapy regimen included conventional cytotoxic drugs (cyclophosphamide (C), paclitaxel (P), fluorouracil (5-FU), epirubicin, Adriamycin (A), etc.). The dosage, time, and frequency of medication, treatment cycles, and all eligible formulations were recorded. Studies that treated the control group with a placebo were not included in our analysis.

#### 2.3.4. Outcome Indicators

The retrieved studies contained primary and secondary indicators, with quality of life being the primary outcome measured in reliable and valid scales. Our investigation identified the following cancer-related symptoms and therapy-related adverse reactions as secondary outcomes: (1) white blood cell (WBC), platelet, and natural killer (NK) cell counts; (2) hormone levels, including estrogen (E_2_) and follicle-stimulating hormone (FSH); (3) immune function markers; (4) body mass index (BMI); (5) incidence of adverse reactions (including gastrointestinal and cardiac dysfunctions and their development related to cancer); (6) tumor markers; (7) safety and tolerance; (8) traditional Chinese medicine syndrome; (9) hair loss; (10) heart function; and (11) toxic side effects.

The quality of life was measured at the time of baseline and after treatment based on reliable and valid scales that have been used around the world with the following scoring and grading tools: Karnofsky Performance Status (KPS), Functional Assessment of Cancer Therapy-Breast (FACT-B), the European Organization for Research and Treatment of Cancer Quality of Life Questionnaire-Core 30 (EORTC QLQ-C30), and the European Organization for Research and Treatment of Cancer Breast Cancer-Specific Quality of Life Questionnaire (EORTC QLQ-BR23).

Each scale had their own total score, with a higher value indicating higher QOL. The Karnofsky Performance Status divided the quality of life into 11 levels, ranging from 0 to 100 scores. The FACT-B contained 37 items covering five dimensions: physical well-being (PWB), social/family well-being (SWB), emotional well-being (EWB), functional well-being (FWB), and additional concerns about breast cancer (BCS). The scores for each question ranged from 0 to 4, and total scores were calculated on a scale of 0 to 148. The EORTC QLQ-C30 consisted of 30 items with 5 function subscales (physical, role, emotional, cognitive, and social), nine symptom subscales (fatigue, nausea, pain, dyspnoea, insomnia, appetite loss, constipation, diarrhea, and financial difficulties), and a global health subscale.

In the KPS, an increase of more than 10 points following treatment was considered an improvement in quality of life, an increase of less than 10 points was regarded as stable, while a decrease in points was considered a deterioration of quality of life. EORTC-BR23 consisted of 23 items with two main subscales: “functional scale (8 items)” and “symptoms scale (15 items).” Items were graded by 4-point Likert ranging from “not at all” (1) to “very much” (4).

### 2.4. Data Extraction and Screening

**Figure 1:**
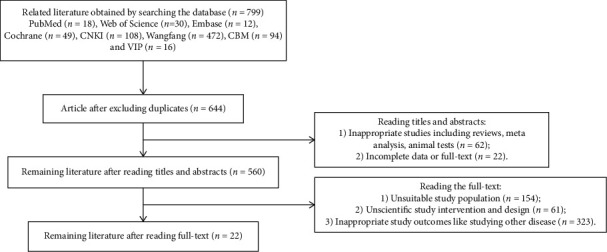
A flowchart depicting the article selection process.

Two researchers (Na Ta and Guo-Hua Gong) independently searched, screened, read, and excluded the irrelevant papers. The selected papers were extracted and imported into NoteExpress for electronic and manual duplicate checks. Publications with inappropriate study designs, incomplete results information, and full text were removed. A full-text screening and data extraction were performed according to the above eligible inclusion and exaction criteria comprising Participants, Interventions, Controls, Outcomes, and Study design framework (PICOS). Any controversial results were cross-checked and discussed with another evaluator until a consistent conclusion and consensus were reached. The following information was extracted from the final eligible articles and recorded in a Microsoft Excel sheet: the name of the first author, year of publication, number of samples in each group, age of the patients, tumor/node/metastasis (TNM) stage, type of intervention, treatment characteristics, such as frequency and duration of intervention, control condition, and outcome indicators, among others. The selection process was shown in a flowchart (see in Figure 1).

### 2.5. Data Analysis

Stata 16.0 software was used to perform meta-analyses. If an outcome indicator was a continuous variable, mean difference (MD) or standardized mean difference (SMD) were effective value selection. For each group, 95% confidence intervals (CIs) were chosen at postintervention compared with baseline. For binary variables, the relative risk ratio (RR) and its confidence interval (CI) were determined. *Homogeneity test*: the statistical heterogeneity of effect estimates across studies was assessed using the *P* value and the *I*^2^ statistic, which estimates the percentage of total variation across studies that can be attributed to heterogeneity rather than to chance. According to the results of quantitatively analysis, *P* ≥ 0.05 and *I*^2^ < 50% were regarded as good agreement, and the fixed effects model (FEM) was used; however, the random effects model (REM) was used for obvious heterogeneity. An effect size of 0.8 was considered large, 0.5 was considered medium, and 0.2 was considered small. *Sensitivity analysis*: because comprehensive factors and variable outcomes can lead to homogeneity, some extreme results of the papers were ruled out before reevaluation and compared with those of meta-analyses before the exclusion to figure out the extent of influence of the excluded studies had on the combined effect value; on other hand, it was regarded as stable and credible.

*Subgroup analysis*: this was performed based on evident homogeneity outcome indicators that could be measured qualitatively. If the necessary data were available, the grouping factor for subgroup analysis would be done for different comprehensive factors (mainly including detailed treatment approaches and secondary indicators for our included studies with the same primary indicator). Stata 16.0 software was used to conduct sensitivity analysis and subgroup analysis and draw a sensitivity analysis chart.

### 2.6. Publication Bias

Publication bias occurred when positive data in similar research papers with statistical significance are more likely to be published on journals in which publication bias was hard to control. The funnel plot method is often used to detect publication bias. Our study applied a funnel plot to detect bias using Stata 16.0 software. Begg's test indicated that there was no publication bias and the funnel plot will be drawn with indexes ≥6 included studies and a two-tailed *P* value of more than 0.05 [30]. If a publication bias existed, the exact reason should be identified. Furthermore, if we were unable to explore the cause of the bias, trimming and filling methods were applied to add or remove parts of required papers for improving the stability.

## 3. Results

### 3.1. Selected Studies Description

A total of 799 related articles that met the search strategy were collected from four Chinese (CNKI: *n* = 108, Wangfang: *n* = 472, CBM: *n* = 94 and VIP: *n* = 16) and four English (PubMed: *n* = 18, Web of Science: *n* = 30, Embase: *n* = 12, Cochrane: *n* = 49) databases. The retrieved articles were recorded in NoteExpress, software that manages documents. The researchers removed 155 duplicated papers through manual and automatic screening. In addition, inappropriate studies including reviews, meta-analysis, and animal tests (*n* = 62) and irrelevant papers with partial data or without full text (*n* = 22) were excluded after reviewing the titles and abstracts. Furthermore, articles that did not meet the inclusion criteria, including articles with unsuitable study populations (*n* = 154), unscientific study interventions and study design (*n* = 61), or inappropriate study outcomes (*n* = 323), were eliminated after reading the full text. Finally, 22 documents were included in the quantitative meta-analysis. The selection process is summarized in Figure 1 using the flow diagram.

### 3.2. Study Characteristics

The selected papers had a total of 1689 patients (intervention group: *n* = 844 and control group: *n* = 845) covering the period from 2005 to 2020. The researched subjects were adult women who had undergone breast surgery due to breast cancer, and the baseline of two groups in each trial, including age and treatment situation, was comparable. The duration of intervention ranged from two weeks to six months. Quality of life was categorized as a primary outcome measurement in all trials. A majority of the studies were conducted in China, and 12 of them were scored using rating scales, while the remainder were measured using ranking scales.  Table 1 shows the details of the study characteristics that were included.

### 3.3. Summary of the Quality and Bias Risk of the Trials

**Figure 2:**
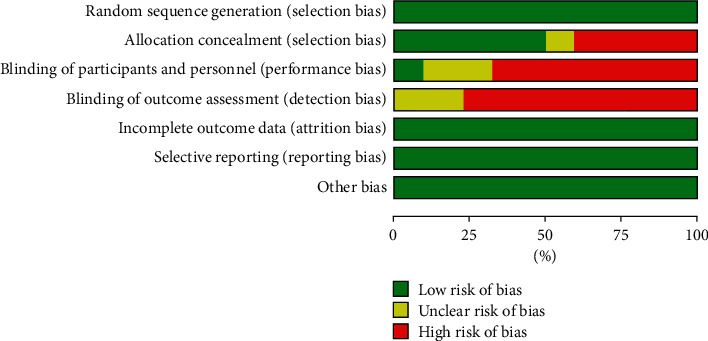
Risk of bias: judgments of the authors on each risk of bias item, which are presented as percentages across all included studies.

**Figure 3:**
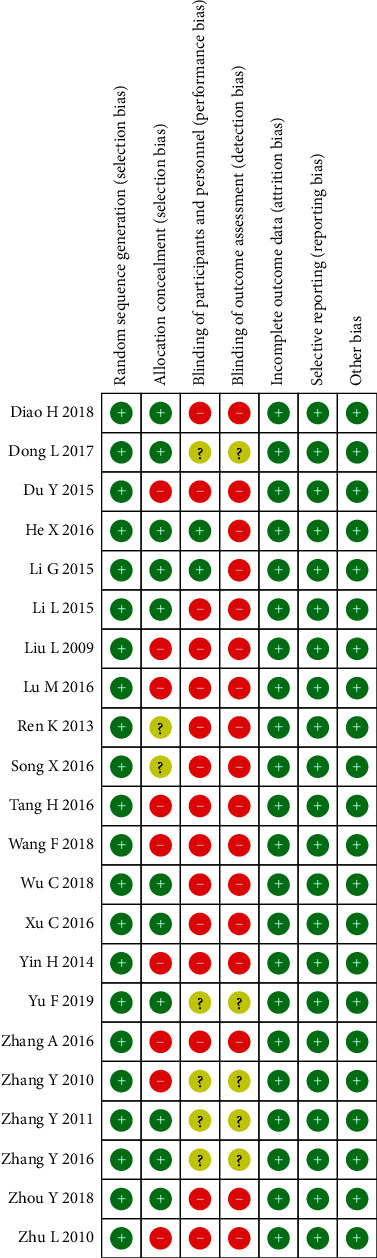
Risk of bias summary: review authors' judgments about each risk of bias item for each included study.

The bias risk of all the included trials was assessed using Cochrane Collaboration's tool. Most of the trials had low risk of bias according to the quality evaluation criteria: 11 studies implemented a specific random scheme, whereas the remaining studies were conducted using a random control test. Most of the trails lacked blinding and allocation concealment. None of the studies adequately reported whether the investigators, patients, or the assessors were blinded. However, two studies reported that they used single blinding. Therefore, outcome assessment blinding was classified as single blinded, nonblinded, or unmentioned. The additional sources of bias in all trails were low due to inclusion criteria. Figures 2 and 3 present a summary and graph showing the risk of bias.

### 3.4. Outcome Measures

#### 3.4.1. The Quality of Life for Rating Scales

**Figure 4:**
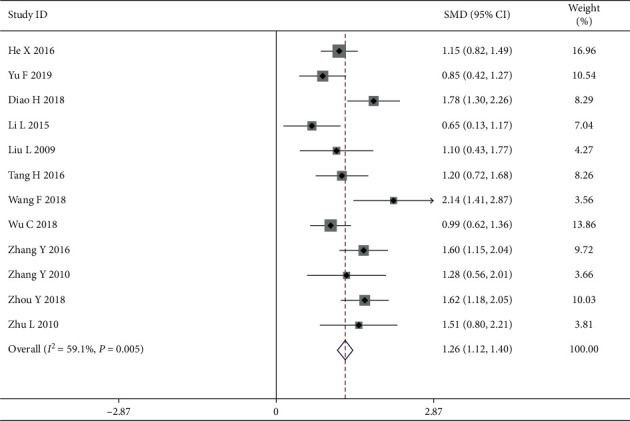
Forest map of included rating scale papers.

Quality of life, which could be divided into two groups and measured by standard and generic scales in the world, was the primary outcome measure for all the included articles. A total of 13 studies, involving 987 patients (492 patients in the intervention group and 494 patients in the control group), used rating scales to measure the QOL. In comparison to the control group, the meta-analyses indicated that combining TCM with CWM treatment improved the QOL (SMD = 1.29, 95% CI (1.07, 1.52)) (Figure 4). There was obvious heterogeneity (*P*=0.01, *I*^2^ = 59.1%), and therefore the random effects model was used to analyze the data. The causes of the observed heterogeneity were further investigated using subgroup and sensitivity analyses. To examine the origin of heterogeneity, some indices can be used to distinguish between subgroups of breast cancer patients with varying QOL.

#### 3.4.2. Subgroup Analysis for Rating Scales

**Figure 5:**
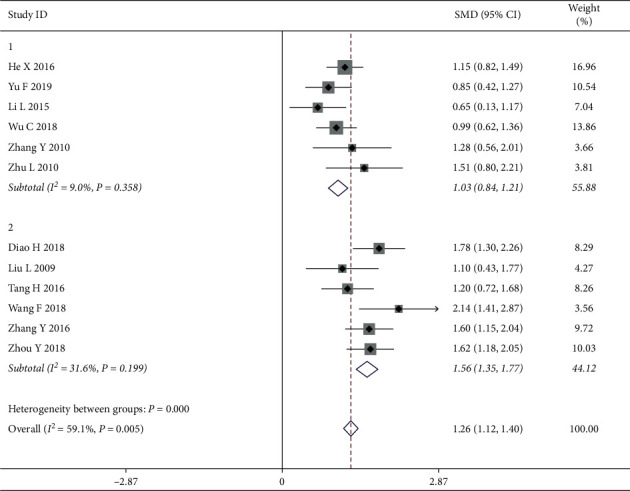
Forest map of rating for subgroups analysis on the adverse reactions.

*(1) The Adverse Reaction*. In our study, the adverse reaction was considered the main effect, and the two subgroups had a high degree of heterogeneity (Group 1: with adverse reaction; Group 2: without adverse reaction), indicating that they were extremely heterogeneous. There was no heterogeneity within each subgroup as shown by an effective value of 1.03 (*Z* = 10.89, *P* < 0.05; Group 1: *I*^2^ = 9.0%, *P*=0.258, SMD = 1.03; and Group 2: *I*^2^ = 31.6%, *P*=0.199, SMD = 1.56), suggesting that the adverse reaction as a treatment secondary indicator may influence the heterogeneity and reduce the QOL among breast cancer patients. In addition, sensitivity analysis was performed and verified by sequentially omitting each study to examine the robustness of the primary outcome. The results of the subgroup analysis can be found in Figure 5.

**Figure 6:**
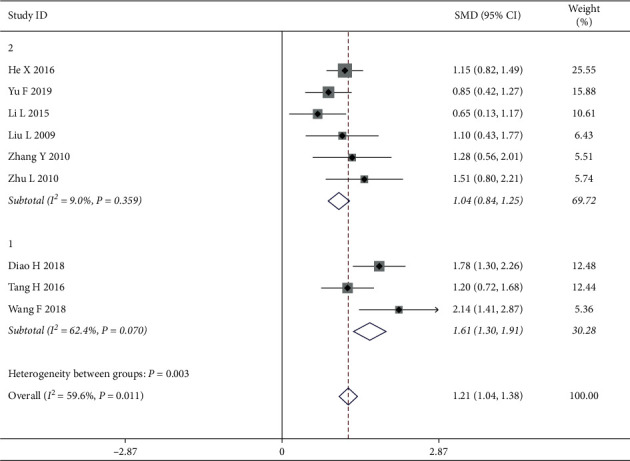
Forest map of rating for subgroups analysis on chemotherapy with epirubicin.

*(2) Chemotherapy with Epirubicin*. Furthermore, the study analyzes the causes of the observed heterogeneity by distinguishing between subgroups with CWM treatment methods. In our study, 9 articles were reported to be treated with chemotherapy among which 3 showed application of epirubicin. The analyzed result showed that the application of epirubicin was considered the factors to influence the heterogeneity, and the two subgroups had a high degree of heterogeneity (Group 1: application of epirubicin; Group 2: nonapplication of epirubicin) with *I*^2^ = 59.6%, *P*=0.011. There was no heterogeneity within each subgroup as shown by an effective value of 1.21 (*Z* = 19.80, *P* < 0.05; Group 1: *I*^2^ = 62.4%, *P*=0.070, SMD = 1.61; and Group 2: *I*^2^ = 9.0%, *P*=0.359, SMD = 1.04), suggesting that the application of epirubicin may influence the heterogeneity and have higher QOL than the chemotherapy without epirubicin among breast cancer patients. The results of the subgroup analysis can be found in Figure 6.

#### 3.4.3. Secondary Results for Rating Scales

**Figure 7:**
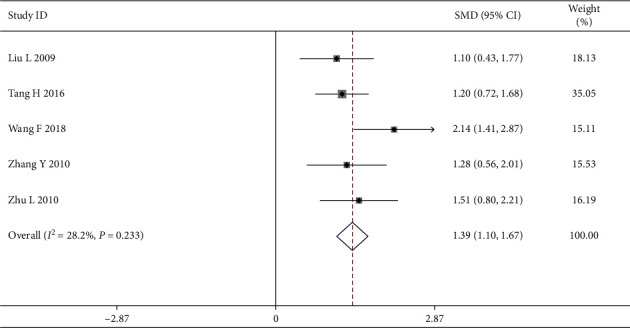
Forest map of included rating scale papers on tumor markers.

*(1) Tumor Markers*. Five articles of changes in tumor makers that are associated with CEA and CA153 were reported. We discovered that there existed relatively small heterogeneity (*I*^2^ = 28.2, *P*=0.233), and therefore the fixed effects model was selected for meta-analysis. The patients with tumor markers in the intervention group had better efficiency in QOL than those in the control group, with a statistically significant difference [SMD = 1.39, 95% CI (1.10, 1.67)] (Figure 7).

#### 3.4.4. The Quality of Life for Ranking Scales

**Figure 8:**
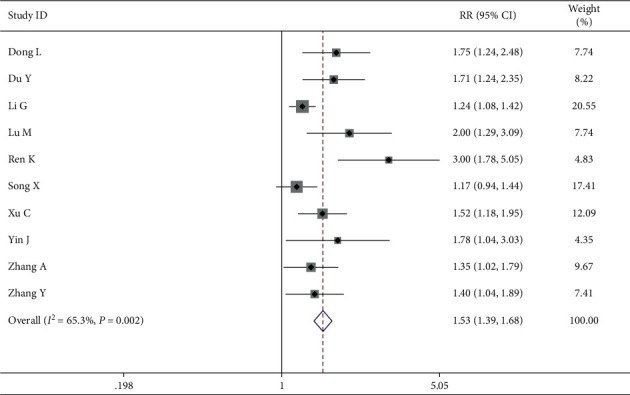
Forest map of included ranking scale papers.

In another group, 10 studies involving 703 patients (352 in the intervention group and 351 in the control group) measured the QOL by ranking scales. The random effects model was used for the data analysis based on the heterogeneity test, which indicated strong heterogeneity (*P*=0.002, *I*^2^ = 65.3%). The meta-analyses revealed that improving the QOL in the intervention group was more successful than in the control group when treating breast cancer (RR = 1.53, 95% CI (1.39 1.68)) (Figure 8). The results were further analyzed using subgroups or trimming and supplementation to reduce heterogeneity.

#### 3.4.5. Subgroup Analysis for Ranking Scales

**Figure 9:**
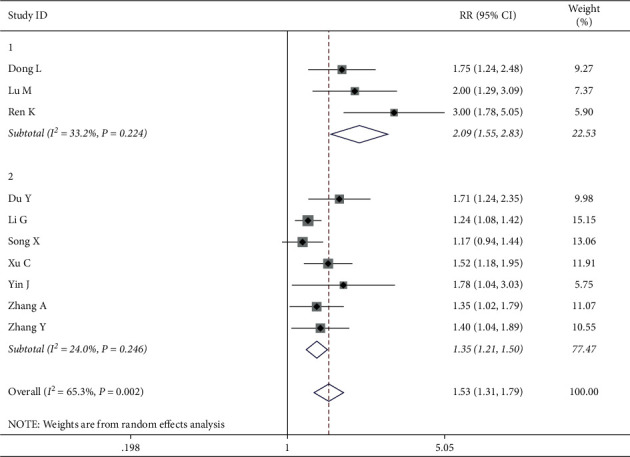
Forest map of ranking for subgroups analysis on chemotherapy with epirubicin.

*(1) Chemotherapy with Epirubicin*. Furthermore, we explored the reason of heterogeneity using subgroup analysis by CWM treatment approaches in which eight of the ten studies treated by chemotherapy used ranking scales. The results revealed that the chemotherapy with epirubicin was the main factor that influenced the heterogeneity, resulting in a high degree of heterogeneity (Group 1: application with epirubicin; Group 2: nonapplication with epirubicin) with *I*^2^ = 65.3%, *P*=0.002. There was no heterogeneity within each subgroup with an effective value of 1.82 (Group 1: *I*^2^ = 33.2%, *P*=0.224, RR = 2.14, and Group 2: *I*^2^ = 29.8%, *P*=0.223, RR = 1.42), indicating that chemotherapy with epirubicin could improve the QOL more effectively among breast cancer patients than the chemotherapy without epirubicin when treating breast cancer (Figure 9).

#### 3.4.6. Secondary Results for Ranking Scales

**Figure 10:**
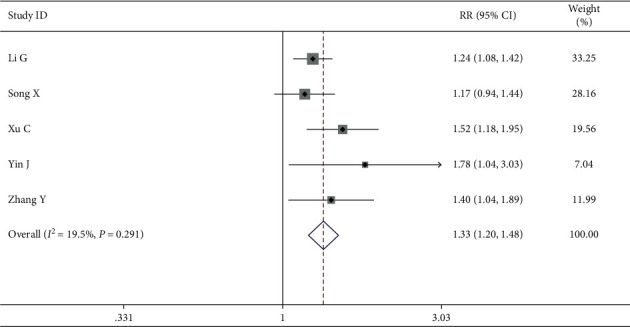
Forest map of included ranking scale papers on gastrointestinal adverse reaction.

*(1) Incidence of Gastrointestinal Adverse Reaction*. A total of 8 trails comprising ranking scales papers reported that the gastrointestinal adverse reaction was a secondary result. The fixed effects model was used to analyze the data because the heterogeneity test showed no heterogeneity (*I*^2^ = 19.5%, *P*=0.291). Meta-analyses indicated that while treating breast cancer, the intervention group had higher efficiency in QOL than the control group among breast cancer patients of gastrointestinal adverse reactions with a statistically significant difference (RR = 1.33, 95% CI (1.20, 1.48)) (Figure 10).

**Figure 11:**
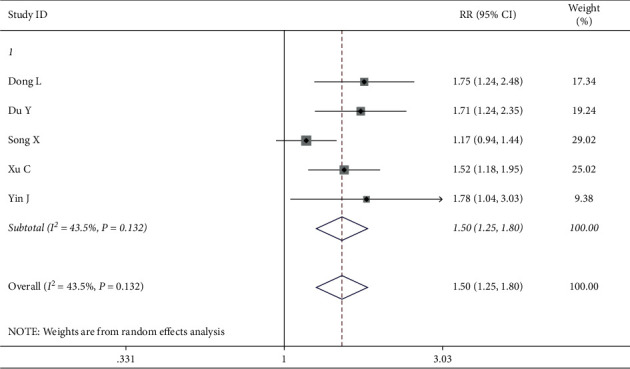
Forest map of included ranking scale papers on the traditional Chinese medicine syndrome.

*(2) Incidence of the Traditional Chinese Medicine Syndrome*. The traditional Chinese medicine syndrome was reported in five papers. The heterogeneity test showed that there was no heterogeneity (*I*^2^ = 43.5%, *P*=0.132) and the fixed effects model was used for data analysis. According to meta-analyses, in the intervention group QOL improved more successfully than in the control group among patients with traditional Chinese medicine syndrome (RR = 1.50, 95% CI (1.28, 1.80)) (Figure 11).

### 3.5. Publication Bias

The publication bias analysis was checked and used to draw a funnel graph using Stata 16.0. Begg's test was performed to measure the publication bias with *P* ≤ 0.05. The trimming and filling method for checking data consistency may reduce publication bias.

**Figure 12:**
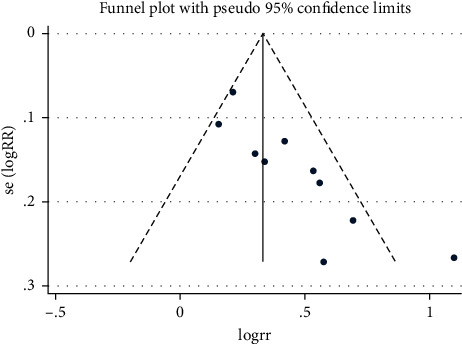
The funnel plot of quality of life by ranking.

**Figure 13:**
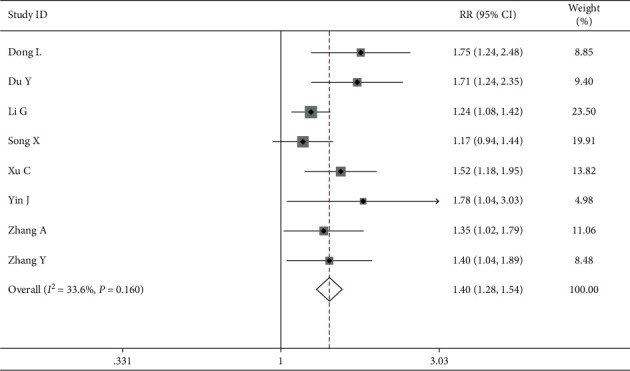
Begg's test of quality of life by ranking after trimming and filling.

**Figure 14:**
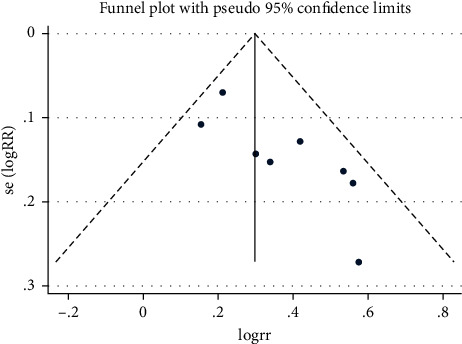
The funnel plot of quality of life by ranking after trimming and filling.

Begg's test revealed suitable publication bias according to rating scales of the QOL especially after subgroup analysis. However, Begg's test and the funnel graph revealed that there existed publication bias (*P*=0.019 < 0.05) for ranking scales (Figure 12). Studies with obvious heterogeneity were removed of Lu M and Ren K (*I*^2^ = 33.6% < 50%, *P*=0.160 > 0.1) and added trails that were similar to the results of Yin J. The above method was called “Trimming and Filling” to stabilize the results and reduce heterogeneity. The detailed analysis results after trimming and filling are shown in Figures 13 and 14.

## 4. Discussion

Breast cancer as the most prevalent malignancy has become increasingly dangerous, ranking first among females worldwide. The CWM, which includes chemotherapy, radiotherapy, and endocrine therapy after surgical treatment, is still considered the most common and active effective treatment for breast cancer. However, the treatment methods and process of CWM may be disfiguring, costly, and accompanied by a range of periodic post-treatment pain, which may cause adverse reactions and side effects that affect QOL even lead to death [31, 32]. Currently, TCM plays a unique role in compensating for inadequacies in CWM treatment. Previous studies had shown that a combined therapeutic approach has the potential function to improve the QOL of breast cancer patients [14, 16]. Statistically significant and clinically meaningful effect sizes had been observed in symptoms of fatigue, emotional status, and sleep difficulty, in favor of TCM [33, 34]. This study systematically reviewed and evaluated 22 trails on the QOL and related outcomes in clinical settings by using integrated TCM and CWM versus using CWM only for the treatment of breast cancer following surgery, thus providing theoretical and scientific evidences and references for filling the gap.

The meta-analysis revealed that several outcomes involving QOL, gastrointestinal adverse reactions, traditional Chinese medicine syndrome, and tumor markers were significantly different between the intervention and control groups. The primary research had revealed that the QOL in the integrated TCM and CWM approach was higher than that in the CWM treatment [30, 35]. Various factors and mechanisms play an important role in the occurrence and development of any tumor, and a single treatment approach could not solve the problem. The part of consistent mechanism among many TCM approaches underlying the treatment of element “Qi” as the foundation of Chinese medicine plays an important role in the treatment of breast cancer for compensating deficiencies of CWM, which may have beneficial psychological, physiological, and immunological effects on improving QOL [29], which was in line with our findings.

In our subgroup analysis, we found that different chemotherapy treatment approaches and adverse gastrointestinal reactions influenced the heterogeneity of measurement QOL. Previous studies showed that all the cancer-related treatment approaches and clinical outcomes had a greater or lesser impact on the QOL [7]. Nowicki and Cortés-Flores have confirmed that patients were treated with different chemotherapeutic drugs and doses and subsequently they experience the different side effects and prognosis [36]. Chemotherapy for cancer patients, such as using fluorouracil (5-FU), often leads to severe diarrhea [37]. In light of this mechanism, our results also demonstrated a favorable tendency in favor of chemotherapy with epirubicin combined with TCM, although our recommendations need to be confirmed by larger randomized controlled trials. Currently, even detail subgroup analysis for improving QOL in the cancer patients is limited and inconsistent, and hence a comprehensive review of our results have been performed; the reasons for this may be unreported outcomes were often nonsignificant and other potential sources of bias existed; in addition, the general concept of TCM was not a single TCM formula or a single herb, which may have inflated the computed heterogeneity [38].

In our study, the secondary outcome revealed that the patients with gastrointestinal adverse reactions, traditional Chinese medicine syndrome, and tumor markers (CEA, CA153) had a significant improvement on QOL and higher efficiency in the integrated treatment group. The heterogeneity of overall quality in included articles was stable. Previous review articles also presented convincing and consistent evidences that TCM, as a supportive assortment for CWM, may decrease adverse reactions and nausea/vomiting symptoms, increase white blood cell count and haemoglobin, and enhanced immunoglobulin in the patients [7, 39]. The mechanism may be that breast cancer patients were prone to coagulation dysfunction due to a large number of fibrin accumulation and platelet aggregation around cancer cells, and therefore, it made the blood present a hypercoagulable state. The function of TCM was to expose cancer cells to be attacked by chemotherapeutic drugs and make them more vulnerable by promoting blood circulation and removing blood stasis, which played the role of increasing efficiency and reducing adverse reactions with chemotherapy [40]. And it is likely that TCM improved the body constitution and immune functions, thus boosting the resistance of patients to adverse reactions [41]. The traditional Chinese medicine syndrome includes emotional depression or irritability, chest and abdominal pain, hot flashes etc. Several studies have indicated that in TCM philosophy, “Qi” as the vital substance constituting the human body had a positive effect of therapy on syndrome performance such as mood improvement and pain reduction [18, 42]. Its explication was consistent with our findings that TCM had a beneficial effect on the improvement of traditional Chinese medicine syndrome. Finally, we discovered that tumor markers were lower in the intervention group than in the control group, implying that the Chinese medicine inhibited the growth and metastasis of tumor cells by enhancing the immune functions [43].

When examining statistical heterogeneity, we examined statistical heterogeneity by subgroup analysis mentioned before, which is also supported by *I*^2^ and *Q* statistics values that indicated more than 50% effect size heterogeneity between studies. In addition, our study included different types of trials employing broader inclusion criteria, synthesizing results including acupuncture, massage, and Tai Chi and Qigong [44]. Many reviews shared common methodological concern that is the moderate to high risk of bias and the heterogeneity of outcome measures––that limit conclusions that could be drawn [45, 46]. Hence, further research is needed to elucidate the role of detailed approaches of TCM and CWM like drugs selection, does, and sequences in the improvement of QOL in women with breast cancer.

## 5. Limitation

The preliminary limitation of this study was publication bias in sample sizes, age of patients, treatment dose, and duration, among others, which may be due to the fact that positive results are more likely to be published during the research publication process. Second, most of the qualified selected clinical trials were conducted in China, and the results failed to prove that this integrated therapy had beneficial effects in other populations, thus limiting the assessment of the accuracy and reliability of the conclusion. Third, QOL as the primary outcome was based on questionnaire-based patient-reported objected results. The future steps need to combine more subjected and precise outcomes. Fourth, the meta-analysis had to be limited to the most frequent outcome measures due to the heterogeneity of outcomes reported in the selected studies. More studies that use the same outcomes to evaluate QOL and the various domains that it can affect should be conducted.

## 6. Conclusion

Although there are some clear limitations to the body of research reviewed in this study, a tentative conclusion can be reached that combining TCM and CWM is an effective therapy for improving QOL and clinical outcomes such as reducing adverse reactions, toxic side effects, the traditional Chinese medicine syndrome, and tumor markers in 1689 breast cancer patients. Based on the results, we should perform long-term and broader trials with a larger and a more diverse sample size. Besides, more and in-depth indexes are required for further verification in the future. This study serves as a resource for breast cancer patients seeking more suitable therapy options and better treatment outcomes.

## Figures and Tables

**Table 1:** The details of the study characteristics.

No.	Author year	Experiment group size (EG)	Control group size (CG)	Age (median or mean and standard deviation) (year)	Is the baseline consistent?	Course of intervention (days)	Intervention group	Control group	Primary outcome evaluation scales (QOL)	Secondary outcomes
1	He X 2016	79	81	EG: 46.82 ± 7.19	Yes	21 d; 9 periods	AF (ADM+5-FU)/AC (ATM + CTX) chemotherapy and radiotherapy and Chinese herbal medicine; A: 50 mg/m^2^, F: 500 mg/m^2^; A: 60 mg/m^2^, C: 600 mg/m^2^	Chinese herbal medicine 400 ml/d	FACT-B	1, 5, 9 11
				CG: 47.17 ± 8.28						

2	Yu F 2019	46	47	EG: 52.40 ± 10.50	Yes	21 d; 6 periods	AD (D: docetaxel) chemotherapy and Xiaoaiping; A: 50 mg/m^2^, D: 75 mg/m^2^	Xiaoaiping 7.2 g/d	FACT-B	1, 5, 9
				CG: 51.50 ± 8.60						

3	Diao H 2018	47	47	EG: 51.68 ± 9.36	Yes	21 d; 6 periods	CEF (E: epirubicin) chemotherapy and Shenqi Fuzheng injection; C: 500 mg/m^2^, E: 100 mg/m^2^, F: 500 mg/m^2^	Shenqi Fuzheng injection 250 ml/d	QLQ-BR53	1, 3
				CG: 52.94 ± 10.14						

4	Li L 2015	30	30	EG: 51.64 ± 10.29	Yes	21 d; 4 periods	TAC (T: TAX) chemotherapy and self-made Chinese herbal medicine; T: 75 mg/m^2^, A: 50 mg/m^2^, C: 500 mg/m^2^	Self-made Chinese herbal medicine	KPS	5
				CG: 49.66 ± 8.58						

5	Liu L 2009	20	20	EG: 61.45 ± 5.24	Yes	28 d; 2 periods	TP (P: DDP) chemotherapy and replenishing Qi and nourishing Yin recipe; T: 60 mg/m^2^, P: 60–100 mg/m^2^	Replenishing Qi and nourishing Yin recipe 200 ml/d	KPS	6, 7, 8
				CG: 61.05 ± 4.48						

6	Tang H 2016	40	39	EG: 47.50 ± 6.90	Yes	21 d; 4 periods	CEF chemotherapy and replenishing Qi and nourishing Yin recipe; C: 500 mg/m^2^ , E: 60 mg/m^2^, F: 500 mg/m^2^	Replenishing Qi and nourishing Yin recipe 150 ml/d	QLQ-C30	1, 6, 8
				CG: 48.10 ± 6.30						

7	Wang F 2018	23	23	EG: 40.36 ± 1.28	Yes	21 d; 3 periods	TP chemotherapy and self-made nourishing Yin recipe; T: 60 mg/m^2^, E: 75 mg/m^2^	Self-made nourishing Yin recipe 150 ml/d	KPS	6
				CG: 41.03 ± 1.18						

8	Wu C 2018	63	63	EG: 44.02 ± 5.16	Yes	3 months	Tamoxifen citrate 20 mg/d and cantharidin capsules	Cantharidin capsules 1.5 g/d	QOL-C3	3, 5
				CG: 44.16 ± 5.19						

9	Zhang Y 2016	52	52	EG: 52.70 ± 5.60	Yes	6 months	Tamoxifen citrate 60 mg/d and Tiaoqi Deji Fang	Tiaoqi Deji Fang 400 ml/d	QLQ-BR53	2, 8
				CG: 53.40 ± 5.10						

10	Zhang Y 2010	18	18	EG: 57.63 ± 8.42	Yes	28 d; 2 periods	TD chemotherapy and replenishing Qi and nourishing blood recipe; T: 60 mg/m^2^, D: 60–100 mg/m^2^	Replenishing Qi and nourishing blood recipe 300 ml/d	FACT-B	5, 6, 7, 8, 10
				CG: 56.69 ± 8.59						

11	Zhou Y 2018	54	54	EG: 68.18 ± 3.62	Yes	6 months	LENM (L: letrozole; E: exemestane; N: nolvadex; M: megestrol) chemotherapy and Yi Wenyang prescription; L: 2.5 mg/d, E: 25 mg/d, T: 20 mg/d, M: 160 mg/d	Yi Wenyang prescription 400 ml/d	QLQ-BR53	-
				CG: 57.27 ± 10.76						

12	Zhu L 2010	20	20	EG: 52.5 ± 8.91	Yes	21 d; 2 periods	AT chemotherapy and self-made different power elimination formulas; A: 60 mg/m^2^, T: 175 mg/m^2^	Self-made different power elimination formulas 300 ml/d	KPS	1, 5, 6, 9, 11
				CG: 50.25 ± 12.58						

13	Dong L 2017	30	30	EG: 51.68 ± 9.36	Yes	21 d; 3 periods	CED chemotherapy and nourishing spleen and kidney recipe; D: 75 mg/m^2^, E: 50 mg/m^2^, C: 500 mg/m^2^	Nourishing spleen and kidney recipe 200 mg/d	KPS	7, 8, 10
				CG: 52.94 ± 10.14						

14	Du Y 2015	30	30	EG: 30.00–69.00	Yes	28 d; 4 periods	CDF chemotherapy and modified Xiaoyao San; C: 500 mg/m^2^ , D: 50 mg/m^2^ , F: 500 mg/m^2^	Modified Xiaoyao San	KPS	8, 11
				CG: 29.00–70.00						

15	Li G 2015	52	52	EG: 58.5 ± 6.57	Yes	28 d; 6 periods	Letrozole: 2.5 g/d & lychee saponins	Lychee saponins 6 tips/d	KPS	1, 2, 3, 5
				CG: 57.8 ± 6.55						

16	Lu M 2016	45	45	EG and CG: 30.00–60.00	Yes	21 d; 2 periods	CEF chemotherapy and Huaier granules; C: 60 mg/m^2^ E: 100 mg/m^2^ F: 60 mg/m^2^	Huaier granules 60 g/d	KPS	1, 3
17	Ren K 2013	32	32	EG and CG: 28.00–79.00	Yes	21 d; 6 periods	CEF chemotherapy and Kanglaite soft capsule C: 500 mg/m^2^ E: 75 mg/m^2^ F: 500 mg/m^2^	Kanglaite soft capsule 10.8 g/d	KPS	4, 9
18	Song X 2016	50	50	EG: 35.00–62.00	Yes	21 d; 4 periods	ACT chemotherapy and Chaihu Shugan powder; A: 60 mg/m^2^, C: 600 mg/m^2^, T: 75 mg/m^2^	Chaihu Shugan powder 500 ml/d	KPS	5, 7, 8, 10
				CG: 36.00–65.00						

19	Xu C 2016	40	40	EG: 49.31 ± 5.28	Yes	28 d; 6 periods	ACF chemotherapy and nourishing Qi and spleen decoction; A: 60 mg/m^2^, C: 600 mg/m^2^, F: 500 mg/m^2^	Nourishing Qi and spleen soup 180 ml/d	KPS	1, 5, 8
				CG: 49.57 ± 6.35						

20	Yin H 2014	20	20	EG: 55.56 ± 5.64	Yes	21 d; 3 periods	CD chemotherapy and Ruyan Xiaoji Fang; C: 600 mg/m^2^, D: 75 mg/m^2^	Ruyan Xiaoji Fang	KPS	1, 5, 6, 8
				CG: 53.89 ± 5.67						

21	Zhang A 2016	30	30	EG: 52.70 ± 11.40	Yes	21 d; 6 periods	ACD chemotherapy and self-made Fuzheng negative soup; A: 50 mg/m^2^, C: 50 mg/m^2^, D: 50 mg/m^2^	Self-made Fuzheng negative soup	KPS	-
				CG: 52.40 ± 10.20						

22	Zhang Y 2011	23	22	EG: 51.24 ± 7.80	Yes	21 d; 2 periods	CFP chemotherapy and Fuzheng Jiedu Quyu recipe; C: 500 mg/m^2^, F: 40 mg/m^2^, P: 400 mg/m^2^	Fuzheng Jiedu Quyu recipe 250 ml/d	KPS	1, 3, 5, 6, 7
				CG: 48.29 ± 7.26						

*Notes*. 1. Intervention group: A = Adriamycin; C = cyclophosphamide; D = docetaxel; E = epirubicin; F = fluorouracil; L = letrozole; M = megestrol; P = paclitaxel; T = tamoxifen. 2. Secondary outcomes: 1. white blood cell (WBC), platelet, and natural killer (NK) cell counts; 2. hormone levels, including estrogen (E_2_) and follicle-stimulating hormone (FSH); 3. immune function markers; 4. body mass index (BMI); 5. incidence of adverse reactions (including gastrointestinal and cardiac dysfunction and their development related to cancer); 6. tumor markers; 7. safety and tolerance; 8. traditional Chinese medicine syndrome; 9. hair loss; 10. heart function; and 11. toxic side effect.

## Data Availability

All the data supporting this meta-analysis are from previously reported studies and data sets, which have been cited. The processed data are available from the corresponding author upon request.

## Abbreviations

TCM: Traditional Chinese medicine
CWM: Conventional Western medicine
QOL: Quality of life
RCT: Randomized controlled trial
CNKI: Chinese National Knowledge Infrastructure
CBM: China Biology Medicine Disc (SinoMed)
VIP: Chinese Scientific Journal Database
Wanfang Data: Wanfang Data knowledge service platform
MD: Mean difference
SMD: Standard mean difference
RR: Relative risk
CI: Confidence interval
EORTCQLQ-C30: The European Organization for Research and Treatment of Cancer Quality of Life Questionnaire-Core 30
EORTC QLQ-BR23: The European Organization for Research and Treatment of Cancer Breast Cancer-Specific Quality of Life Questionnaire
KPS: Karnofsky Performance Status
SF-36: The MOS 36-Item Short-Form Health Survey
FACT-B: Functional Assessment of Cancer Therapy-Breast
PWB: Physical well-being
SWB: Social/family well-being
EWB: Emotional well-being
FWB: Functional well-being
BCS: Additional concerns about breast cancer
TCM-FEMT: Traditional Chinese Medicine Five-Element Music Therapy
PRISMA: Preferred Reporting Items for Systematic Reviews and Meta-Analysis
WBC: White blood cell
NK: Natural killer
E_2_: Estrogen
FSH: Follicle-stimulating hormone
BMI: Body mass index
PICOS: Participants, Interventions, Controls, Outcomes, and Study design
FEM: Fixed effects model
REM: Random effects model.
